# Risky Driving Behaviours and Their Association With Fracture Injury Severity in Road Traffic Accident Patients

**DOI:** 10.7759/cureus.99258

**Published:** 2025-12-15

**Authors:** Zeeshan Umar, Ansar Mehmood, Daniyal Azam, Mushtaq Ahmed, Abdullah Bin Naeem, Muhammad Haseeb Amjad, Sami Merie, Saba Qayum, Anas J Khan, Sewar S Omar, Ali Asif

**Affiliations:** 1 Emergency Medicine, Pak Emirates Military Hospital (PEMH), Rawalpindi, PAK; 2 Trauma and Orthopaedics, Letterkenny University Hospital, Letterkenny, IRL; 3 Trauma and Orthopaedics, Services Hospital, Lahore, PAK; 4 Trauma and Orthopaedics, Shaheed Mohtarma Benazir Bhutto Medical College, Karachi, PAK; 5 Orthopaedics, Pakistan Institute of Medical Sciences, Islamabad, PAK; 6 Trauma and Orthopaedics, Avicenna Medical College and Hospital, Lahore, PAK; 7 Trauma and Orthopaedics, Queen Elizabeth Hospital, London, GBR; 8 Accident and Emergency, Rural Health Center, Dina, PAK; 9 Anaesthesia and Critical Care, Buch International Hospital, Multan, PAK; 10 Medicine and Surgery, Jordan University Hospital, Amman, JOR; 11 Medicine and Surgery, Pakistan Institute of Medical Sciences, Islamabad, PAK

**Keywords:** driver behaviour questionnaire, fracture severity, injury severity score, risky driving behaviour, road traffic injuries

## Abstract

Background: Road traffic injuries (RTIs) are an increasingly significant population health problem in Pakistan. Such accidents cause fractures and are linked to unsafe driving behaviours. Determining the predictors of injury severity through behavioural means can reinforce the need for specific prevention methods. The study aimed to determine the correlation between risky driving behaviours and the severity of injuries in patients with road traffic-related fractures.

Procedures: The cross-sectional study was conducted from July 2024 to May 2025 at tertiary care hospitals in Islamabad, Rawalpindi, and Lahore, Pakistan. A convenience sampling method was used to enrol 400 adult patients with road traffic-related fractures. The data were collected using a structured questionnaire that included demographic information, the Driver Behaviour Questionnaire (DBQ), and the Injury Severity Score (ISS). Statistical tests were Pearson correlation, independent t-tests, one-way analysis of variance (ANOVA), and multiple linear regression.

Results: Of 400 respondents, 210 (53%) were male, and 190 (47%) were female, with the largest proportion aged 18-24 years (n = 180; 45%). DBQ and ISS scores were positively correlated (r = 0.612, p < 0.001). There were higher mean DBQ (M = 172.8 ± 13.2) and ISS (M = 24.1 ± 4.5) scores in males compared to females (M = 163.4 + 12.7 and M = 21.6 + 4.0, respectively; p < 0.001). Regression analysis showed that the risky driving behaviour and older age were associated with increased severity of injuries, whereas female gender, a valid driving licence, and a higher driving experience were protective (p < 0.01).

Conclusion: Risky driving behaviours have a significant impact on increasing the severity of injuries in road traffic fracture patients. Trauma-related outcomes may be associated with safer driving behaviours, adherence to licensing rules, and appropriate driver training.

## Introduction

Road traffic injuries (RTIs) are a global health concern, with more than 85% of deaths and 90% of disability-adjusted life years (DALYs) taking place in low- and middle-income countries, which have the weakest health systems available to deal with them [1,2]. Fractures resulting from road traffic accidents are reported to affect 1-2.9 million people per year worldwide, predominantly involving young groups [3].

Epidemiological studies revealed that males of young age were the most affected, motorcycles and head-on collisions were standard mechanisms of trauma, and fractures of the tibia and fibula led amongst all injuries, emphasising the importance of better road safety measures and trauma care [4]. By comparison, elderly patients have an almost doubling of the mortality rate compared to their younger cohorts. They are more likely to sustain chest trauma, while the most common mechanisms of injury involve impacts that often result in fractures of the malar and maxillary bones [5,6].

A recent trauma registry study in Karachi, Pakistan, reported that RTIs accounted for 56% of admitted adult trauma cases, with extremity and head/neck injuries most common and in-hospital mortality of 15% [7]. In addition, a study among adult pedestrians found that higher health literacy was significantly associated with safer pedestrian behaviours, including adherence to traffic rules and safe road-crossing practices, highlighting the importance of targeted interventions to improve road-user safety [8]. Furthermore, a 2024 study found that risky behaviours - including speeding, traffic errors, and non-helmet use - were significant predictors of crashes, particularly among younger riders, those with lower education or income, and unlicensed riders, highlighting the need for targeted safety interventions [9].

The objective of the current study is to examine the relationship between risky driving patterns and the severity of clinical injuries among patients presenting to tertiary care orthopaedic centres with road-traffic-related fractures. The study aims to support preventive interventions and encourage safer driving habits by identifying behavioural risk determinants of more severe injuries in the groups at risk of severe orthopaedic trauma.

Rationale

RTIs have emerged as a significant issue of societal health concern in Pakistan, where the rate of urbanisation and the subsequent growth in vehicle density have caused a growing number of accidents [7]. The lack of implementation of traffic rules is another factor that has added to this burden. RTIs can cause significant physical disability and are associated with considerable social and economic costs for patients, their families, and the healthcare system. Nevertheless, little local evidence exists regarding the role of protective precautions (helmet and seatbelt use) and behavioural determinants in the level of injuries sustained in road traffic accidents. The described associations are crucial in devising specific prevention measures that might alleviate the severity of injuries, enhance the recovery process, and reinforce trauma care practices in Pakistani tertiary care hospitals. Through the study of behavioural and clinical predictors, this research seeks to produce locally applicable evidence that would facilitate safer driving behaviours and better outcomes in orthopaedic traumas.

Objectives

General Objective

The general objective was to examine the association between risky driving behaviours and injury severity among fracture patients in tertiary care orthopaedic hospitals in Pakistan, using a cross-sectional study design.

Specific Objectives

Specifically, the study aimed to quantify injury severity among road traffic fracture patients using the Injury Severity Score (ISS); to assess the relationship between risky driving behaviour, as measured by the Driver Behaviour Questionnaire (DBQ), and injury severity; to describe the socio-demographic characteristics of patients, including age, gender, driving experience, license status, and road user type; and to characterise the overall demographic and occupational profile of patients presenting with road traffic-related fractures in tertiary care hospitals.

## Materials and methods

A cross-sectional study was conducted between July 2024 and May 2025 to investigate the association between risky driving behaviours and injury severity in patients with road traffic-induced fractures. Adults who had road traffic accident-related fractures were taken as study participants from the tertiary care centres of Islamabad, Rawalpindi, and Lahore, Pakistan.

This investigation was based on observational, non-invasive data ascertainment and did not involve diagnostic or laboratory tests. All participants provided informed consent, and participation was voluntary. We employed convenience sampling due to limited resources and time to conduct the study in tertiary care hospitals in Pakistan. This approach enabled recruitment of all eligible and willing patients during the study period, though it may limit generalizability and introduce selection bias. Recruitment was undertaken at multiple tertiary care centres to obtain broader representation from the Pakistani healthcare context.

Sample size and technique

No precise data are available on the total number of individuals with road traffic-related fractures in the Pakistani general population; therefore, the population was assumed to be infinite in our study. The required minimum sample size was calculated as per the formula:

\[n = \frac{Z^2 \cdot p (1 - p)}{d^2}\]

where Z = standard value corresponding to desired confidence level, p is the estimated proportion, and d = margin of error. A 95% confidence level was adopted, with a Z-score of 1.96 and a margin of error of 0.05. Due to a lack of prior reliable estimates of p in the local context, a conservative estimate of p = 0.50 was used to meet the maximum sample size requirement [10]. Based on this approach, the smallest sample size was calculated as 384 participants. To compensate for potential nonresponses and incomplete surveys, additional participants were included, yielding a final sample of 400 respondents.

The stepwise sampling process is as follows: patients presenting with RTA-related fractures were screened for eligibility, approached for informed consent, and enrolled if they met the inclusion criteria. Those who declined to participate or were unable to respond were excluded.

Inclusion criteria

All adult patients (aged 18 years or older) with fractures resulting from road traffic accidents who presented to the selected tertiary care hospitals during the study period were invited to participate. Both male and female patients were included, regardless of vehicle type or fracture location. Participants who provided informed consent and agreed to participate were enrolled in the study.

Exclusion criteria

Exclusion criteria included patients with pathological fractures, non-traffic accident-related fractures (including falls, assaults, and sports-related injuries), and records considered unreliable due to missing essential clinical or demographic information, inconsistent documentation, or ambiguity in fracture details. Patients who were unable to participate in data collection, including those who were critically ill or those who declined to provide informed consent, were not included in the study.

Data collection tools

This investigation employed a structured questionnaire comprising three sets: demographic data (age, sex, and most recent profession), safety/behavioural factors (participation in protection programs), and clinical predictive components of fracture severity/complication. The questionnaire was available only in English and was not professionally translated or culturally adapted. This may have excluded lower-literacy and non-English-speaking participants, potentially introducing systematic measurement bias. The complete questionnaire is available in Appendices A-B for reference.

Demographic information

Demographic and background information were gathered in the first section, as these factors may affect road traffic-related fractures. Data on age, gender, occupation, educational status, and vehicle type were collected. This part enabled subgroup comparisons according to demographic characteristics associated with RTIs in Pakistan.

Driver Behaviour Questionnaire (DBQ)

The DBQ was first developed in 1990 by Reason and his colleagues as a self-reported measure of risky driving behaviours. The complete 50-item version of the DBQ was applied in this study. The tool incorporates three core latent domains: errors (inadvertent errors of perception and judgment), lapses (failures of memory and attention), and violations (intentional breaches of safe driving behaviour). A behaviour is scored for each item on a scale of 1-6, with higher scores reflecting how often this occurs across all items coded together. Subscores and complete scores can be calculated for driving behaviour patterns. The DBQ demonstrates sound internal consistency, with Cronbach's alphas ranging from 0.70 to 0.92 for the scale components [11]. Use of the DBQ in this study was authorised through formal written permission obtained from the original copyright holder, Professor Antony Manstead (see Appendix A).

Injury Severity Score (ISS)

The ISS was used as a standard tool to assess the severity of injuries in victims. It was introduced by Baker et al. in 1974 as a standardised anatomical scoring system for quantifying trauma severity. It is based on the Abbreviated Injury Scale (AIS), which assigns scores of 1 (minor) to 6 (unsurvivable). ISS is derived by squaring the three highest AIS scores from three body regions and summing them, yielding a range of 1-75. Higher scores indicate greater overall injury severity and a higher risk of morbidity and mortality. The ISS has become a standard tool in both trauma research and clinical medicine due to its capacity to provide an objective assessment of injury severity. Although the ISS is not a psychometric scale, it is a well-established anatomical scoring system derived from AIS codes. It has been extensively validated as a predictor of trauma severity and clinical outcomes in hospitalised patients (see Appendix B) [12].

Procedure and ethical considerations

Patients with RTA-related fractures were enrolled from the emergency, orthopaedic, and trauma departments of tertiary care hospitals during the study duration. Demographic, clinical, and accident data were obtained directly from the patients and from their medical records. Complications, including wound infection, delayed union, malunion, neurovascular compromise, surgical site problems, and mortality, were documented during admission and follow-up where possible. For participants who were not proficient in English, the research team assisted to ensure they could understand and complete the questionnaire accurately. All patient data were anonymised using unique codes and securely stored on password-protected systems to maintain confidentiality.

The Institutional Ethics Review Board of the Pakistan Institute of Medical Sciences (PIMS/IRB/2024/274) approved the study, and all procedures in the current investigation adhered to the principles outlined in the Declaration of Helsinki. Voluntary participation, confidentiality, and secure data management were guaranteed, and participants were informed that refusal or withdrawal would not affect their medical treatment.

Statistical analysis

The statistical analysis was conducted with IBM SPSS Statistics for Windows, Version 26 (Released 2018; IBM Corp., Armonk, New York, United States). Demographic characteristics of the participants were described using descriptive statistics and are presented as frequencies (n) and percentages (%). Q-Q plots were used to assess the normality of continuous variables visually. A Pearson correlation analysis was conducted to examine the relationship between the DBQ and the ISS. A t-test was used to compare mean DBQ and ISS scores between male and female participants, and a one-way analysis of variance (ANOVA) was used to compare the mean scores across age groups. A multiple linear regression model assessed predictors of injury severity, including DBQ, age, gender, road user type, license status, and years of driving experience. Regression results are reported in standardised format: unstandardised coefficient (B), standardised beta (β), 95% confidence interval (CI), t-statistic, and p-value. Two-tailed tests were used, with p < 0.05 considered statistically significant.

## Results

Table 1 indicates that out of 400, the majority were young adults (18-24 years; N = 180, 45%) and male (N = 210, 53%). Almost half were married (N = 180, 45%), with most having completed secondary education (N = 151, 38%). The majority resided in semi-urban places (N = 168, 42%) and were currently employed (N = 174, 43%). The most significant proportion of victims was car passengers (N = 118, 29%) and driver or motorcycle/scooter riders (N = 110, 27%). With respect to licensing status, 114 (29%) of drivers were on learner or probationary licenses, and 112 (28%) had no permit. The majority had one to five years of past driving experience (N = 119, 30%). Categorised by smoking status, 178 (45%) were ex-smokers, 146 (36%) smokers and 76 (19%) non-smokers.

**Table 1:** Demographic Characteristics of Participants (N = 400) Values are presented as N (%). No statistical comparisons were performed for demographic variables in this table. f: frequency

Variable	f (N)	%
Age		
18-24 years	180	45
25-34 years	120	30
35-44 years	50	12
45-54 years	25	6
55-64 years	15	4
65 years and above	10	2
Gender		
Male	210	53
Female	190	47
Marital status		
Single	96	24
Married	180	45
Widowed/divorced/separated	124	31
Educational level		
No formal schooling	39	10
Primary	98	24
Secondary	151	38
Higher secondary/intermediate	83	21
Graduate	29	7
Occupation		
Student	74	18
Employed	174	43
Unemployed/housewife	127	33
Retired	25	6
Residence		
Urban	97	24
Semi-urban	168	42
Rural	135	34
Primary road user at the time of the accident		
Driver (car)	53	13
Driver (motorcycle/scooter)	110	27
Passenger (car)	118	29
Passenger (motorcycle/scooter)	61	15
Pedestrian	29	7
Cyclist	16	4
Other	13	3
Driver's license status (if driver)		
Valid license	101	25
Learner/probationary license	114	29
No license	112	28
Not applicable	73	18
Years of driving experience (if driver)		
<1 year	63	16
1-5 years	119	30
6-10 years	115	29
More than 10 years	75	19
Not applicable	28	7
Smoking status		
Never smoked	76	19
Former smoker	178	45
Current smoker	146	36

**Figure 1:**
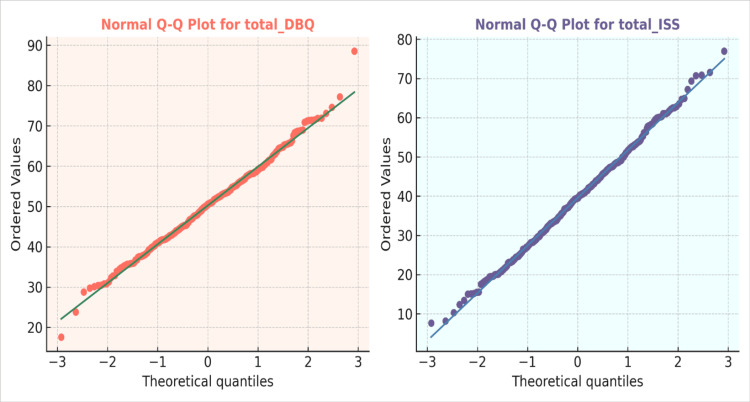
Normal Q-Q Plots of the Total Driving Behaviour Questionnaire (DBQ) and Injury Severity Score (ISS) (N = 400) The figure illustrates normal Q-Q plots for the total DBQ and the total ISS. The data points align closely with the diagonal reference lines, indicating that both variables are generally distributed according to visual inspection.

Figure 1 demonstrates the normal Q-Q plots for the total DBQ and the ISS among 400 participants. Both variables' data points are close to the reference line of the diagonal, which means that the distributions of the DBQ and ISS values are close to normality. The tails only exhibit some slight deviations, which indicate that the data are typically distributed and can be analysed using parametric statistical tests, including correlation, t-tests and ANOVA.

Table 2 shows the correlation between the DBQ and the ISS among 400 participants. There was a significant positive correlation between DBQ scores and ISS (r = 0.612, t(398) = 15.44, p < 0.001), indicating that participants reporting more frequent risky driving behaviours tended to have higher injury severity. These findings suggest that higher levels of dangerous driving behaviours are associated with greater injury severity in road traffic accidents.

**Table 2:** Correlation Between Driving Behaviour Questionnaire (DBQ) and Injury Severity Score (ISS) (N = 400) Values represent Pearson correlation coefficients (r) between continuous variables; ** p < 0.001 was considered statistically significant.

Variables	DBQ	ISS
DBQ	-	-
ISS	r = 0.612, t(398) = 15.44, p < 0.001^**^	-

Table 3 indicates that there are variations in gender between driving behaviour and severity of injury among the participants (N = 400). Participants of male sex scored higher in DBQ (M = 172.80 ± 13.20) compared to their female counterparts (M = 163.40 ± 12.70), t (398) = 4.92, p < 0.001, which is a large effect size (Cohen d = 0.73). Equally, ISS (M = 24.10 + 4.50) were also higher among males (M = 21.60 + 4.00), t (398) = 5.64, p < 0.001, with a medium effect size (d = 0.59). These results show that male participants had a higher risk of driving behaviour and had more serious injuries than female participants.

**Table 3:** Independent Samples t-Test Comparing Male and Female Participants on Driving Behaviour and Injury Severity Scores (N = 400) Values are presented as mean ± standard deviation (SD); Independent samples t-tests were conducted to compare participants of both genders; Group sizes are shown as N (%); Reported statistics include p-values, t-values, 95% confidence intervals (CIs), and effect sizes (Cohen's d); ** A p-value < 0.001 was considered statistically significant. LL: lower limit; UL: upper limit

Variable	Male (N = 210; 53%) M ± SD	Female (N = 190; 47%) M ± SD	t	p	Cl 95% LL	UL	Cohen's D
Driving Behaviour Questionnaire (DBQ)	172.80 ± 13.20	163.40 ± 12.70	4.92	<0.001^**^	5.65	13.15	0.73
Injury Severity Score (ISS)	24.10 ± 4.50	21.60 ± 4.00	5.64	<0.001^**^	1.63	3.37	0.59

Table 4 shows the comparison of the DBQ and ISS in various age groups (N = 400). There was a significant variation in the scores of DBQ between age groups, F(5,394) = 26.84, p < 0.001, η^2^ = 0.25, showing that younger participants (18-24 years: M = 173.8, 12.5) had higher risky driving behaviours than older participants (65+ years: M = 150.3, 10.8). Likewise, ISS also had a considerable difference among age groups, F(5,394) = 21.67, p < 0.001, η^2^ = 0.22, with older participants (65+ years: M = 28.1 ± 3.8) exhibiting a higher level of injuries than younger ones (1824 years: M = 20.6 ± 4.2). These findings support the idea that risky driving behaviour is more prevalent among younger drivers, but the severity of injuries tends to increase with age.

**Table 4:** One-Way ANOVA Comparing Driving Behaviour Questionnaire (DBQ) and Injury Severity Score (ISS) Across Age Groups (N = 400) Data are presented as mean ± standard deviation (M ± SD); Group sizes are shown as N (%). One-way analysis of variance (ANOVA) was conducted to examine the effect; ** All comparisons were significant at p <0.001; η^2^ represents the partial eta-squared effect size.

Variable	18-24 years (N = 180; 45%); M ± SD	25-34 years (N = 120; 30%); M ± SD	35-44 years (N = 50; 12%); M ± SD	45-54 years (N = 25; 6%); M ± SD	55-64 years (N = 15; 4%); M ± SD	65+ years (N = 10; 2%); M ± SD	F(5,394)	p	η^2^
DBQ	173.8 ± 12.5	168.6 ± 11.8	159.4 ± 13.1	151.7 ± 12.4	148.9 ± 11.2	150.3 ± 10.8	26.84	<0.001^**^	0.25
ISS	20.6 ± 4.2	21.4 ± 4.5	23.1 ± 4.6	25.2 ± 4.3	26.7 ± 4.1	28.1 ± 3.8	21.67	<0.001^**^	0.22

Table 5 presents the results of a multiple linear regression predicting ISS from driving behaviour, age, gender, and other driving-related factors among 400 participants. Higher DBQ scores, reflecting more frequent risky driving behaviours, were significantly associated with greater injury severity (B = 0.118, β = 0.342, p < 0.001). Age was also positively associated with ISS (B = 0.842, β = 0.216, p < 0.001). In contrast, female gender (B = -1.987, β = -0.182, p = 0.002), being a primary road user at the time of the accident (B = -0.412, β = -0.138, p = 0.002), having a valid driver’s license (B = -0.573, β = -0.121, p = 0.001), and greater driving experience (B = -0.238, β = -0.164, p = 0.001) were associated with lower injury severity. These findings suggest that more frequent risky driving and older age are linked with higher injury severity, whereas female gender, licensing, primary road user status, and driving experience appear protective.

**Table 5:** Multiple Linear Regression Predicting Injury Severity Score (ISS) From Driving Behaviour, Age, Gender, and Driving-Related Factors (N = 400) Multiple linear regression was conducted to identify predictors. Values include unstandardized coefficients (B), 95% confidence intervals (CIs), standard error (SE), standardised beta coefficients (β), and p-values; ** p < 0.01, *** p < 0.001 were considered statistically significant. LL: lower limit; UL: upper limit

Predictor	B	SE	β	t	p	95% CI LL	95% CI UL
Constant (ISS)	8.412	2.846	-	2.956	0.003^**^	2.814	14.010
Total Driving Behaviour Questionnaire (DBQ)	0.118	0.021	0.342	5.619	<0.001^***^	0.077	0.159
Age	0.842	0.205	0.216	4.110	<0.001^***^	0.438	1.246
Gender (1 = female)	-1.987	0.638	-0.182	-3.115	0.002^**^	-3.241	-0.733
Primary road user at the time of the accident	-0.412	0.129	-0.138	-3.196	0.002^**^	-0.666	-0.158
Driver's license status (if driver)	-0.573	0.178	-0.121	-3.221	0.001^**^	-0.924	-0.222
Years of driving experience (if driver)	-0.238	0.069	-0.164	-3.448	0.001^**^	-0.374	-0.102

## Discussion

The current study examined the association between risky driving behaviours and the injury severity among patients with road traffic-related fractures in tertiary care hospitals in Pakistan. In our research study, there was a strong positive relationship between risky driving behaviour and injury severity. A study reported similar results and showed that dangerous driving behaviours like speeding and racing were linked with increased risk of motor vehicle injuries [13].

Male participants in our study demonstrated substantially greater risky driving behaviour than female participants, as indicated by a higher DBQ score. This result aligns with an earlier study, which also found that male drivers reported engaging in risky driving more than their female counterparts, as male drivers were more likely to experience positive affect and a lesser degree of risk perception when they did so [14]. In our research, males had a considerably higher score of injury severity compared to females, indicating that male drivers are at a greater risk of severe injuries. Previous studies have reported similar findings, noting significant gender differences in injury severity, with males experiencing more severe crash outcomes than their female counterparts [15].

The younger drivers (18-24 years) in our study had much higher DBQ scores, indicating they were more engaged in risky driving than older age groups. This is in line with prior research, which found that young drivers, particularly male drivers, were more likely to undertake risky manoeuvres on the road than their older counterparts. This reinforces the role of age as a risk factor associated with driving [16]. Conversely, older drivers (65+ years) had higher ISS scores than younger drivers, indicating that injury severity increases with age. This observation aligns with a study, which stated that more serious or fatal injuries were more likely to affect older drivers in a variety of crash situations, which supports the role of age in the severity of injuries [17].

Regression analysis supported these associations, showing that higher DBQ scores, male gender, and advancing age were independently associated with higher injury severity (Table 5) [13,15,17]. Passengers in our study had less ISS than did drivers, in line with a previous cohort study that indicated that occupants in the rear seat were less likely to suffer severe trauma and fatal injuries than those in the driver seat [18].

Injury severity was lower among drivers with a valid license compared to those without a license or with only a learner permit, as shown in Table 5. This finding aligns with prior research indicating that drivers without valid licenses are more likely to be involved in road crashes and to exhibit high-risk driving behaviours. These results suggest that licensing and adequate driver education have a protective effect on injury severity [19]. Lastly, greater driving experience was associated with lower injury severity, with each additional year of driving experience linked to a 0.238-point decrease in ISS (B = -0.238, β = -0.164, p = 0.001). This is supported by research showing that experience can play a protective role in improving vehicle control, anticipation, and decision-making during accidents [20].

Overall, these results highlight the importance of interventions targeting high-risk drivers, particularly young and male drivers, and emphasise the need for licensing, driver education, and strategies to reduce road safety risks for all occupants.

Some limitations should be acknowledged. First, the study used a cross-sectional design, which does not allow causal inferences regarding the relationship between risky driving behaviour and injury severity. Second, convenience sampling was employed, which may introduce selection bias and limit the generalisability of findings to the broader population of road users in Pakistan. Third, all behavioural and injury data were collected using structured questionnaires: the DBQ for risky driving behaviours and the ISS for injury severity. The DBQ was available only in English and was not culturally adapted or validated for the local population, likely excluding participants with lower literacy or non-English speakers. Similarly, although the ISS is a standardised anatomical scoring tool, the procedures for scoring, personnel training, and inter-rater reliability were not documented. These factors may have introduced systematic measurement bias, potentially skewing the results toward more educated or urban participants and inconsistently scored injuries. Fourth, all behavioural data were self-reported, which may be subject to recall and social desirability biases. Fifth, the study included only patients with fractures admitted to tertiary care hospitals, which may limit generalisability to all RTI victims or to non-fracture cases. Lastly, external variables such as road conditions, vehicle type, weather, and protective gear use at the time of the accident were not considered, which may have influenced injury outcomes.

Future directions

Future studies should use longitudinal or case-control designs to examine how driving behaviours correlate over time and contribute to injury risk. Improving reliability and interpretability requires modifying and validating culturally relevant behavioural measures, such as the DBQ, and translating them into local languages. Additionally, incorporating objective data, such as police crash records, vehicle safety information, and on-site environmental assessments, would provide a more detailed understanding of injury determinants. Finally, intervention-based studies assessing the effectiveness of behavioural modification campaigns, licensing reforms, and social sensitisation programs are warranted to guide targeted prevention strategies in Pakistan's rapidly urbanising context.

## Conclusions

This research indicates a positive association between risky driving behaviours and the severity of injuries inflicted during road traffic-related fractures. Higher risk of unsafe driving was observed among younger drivers, whereas greater injury severity was associated with older age, as well as male gender and unlicensed or inexperienced driving. In contrast, the female gender, valid licensing, and higher driving experience had a protective impact. These results highlight the importance of increased efforts in road safety education, tighter licensing regulations, and interventions targeting high-risk groups to improve their behaviour. Evidence-based preventive measures might be very critical in minimising the physical, social, and economic costs of RTIs in Pakistan.

## Appendices

Appendix A 

**Table 6:** Driver Behaviour Questionnaire (DBQ) Developed by Reason and his colleagues (1990) [11].

Items	Responses
Attempt to drive away from traffic lights in third gear	-
Check your speedometer and discover that you are unknowingly travelling faster than the legal limit	-
Lock yourself out of your car with the keys still inside	-
Become impatient with a slow driver in the outer lane and overtake on the inside	-
Drive as fast along country roads at night on dipped lights as on full beam	-
Attempt to drive away without first having switched on the ignition	-
Drive especially close or „flash‟ the car in front as a signal for that driver to go faster	-
Forget where you left your car in a multi-level car park	-
Distracted or preoccupied, realise belatedly that the vehicle ahead has slowed	-
Intend to switch on the windscreen wipers, but switch on the lights instead, or vice versa	-
Turn left onto a main road into the path of an oncoming vehicle	-
Misjudge your gap in a car park and nearly (or actually) hit adjoining vehicle	
‟Wake up‟ to realise that you have no clear recollection of the road travelled	-
Miss your exit on a motorway and have to make a lengthy detour	-
Forget which gear you are currently in and have to check with your hand	-
Stuck behind a slow-moving vehicle on a two-lane highway, try to overtake in risky circumstances	-
Intending to drive to destination A, „wake up‟ to find yourself on route to B	-
Take a chance and cross on lights that have turned red	-
Angered by another driver's behaviour, give chase to confront him/her	-
Try to overtake without checking your mirror and get hooted at by car behind	-
Deliberately disregard the speed limits late at night or very early in the morning	-
Forget when your road tax/insurance expires and discover you are driving illegally	-
Lost in thought, forget that your lights are on full beam until „flashed‟ by other motorists	-
On turning left, nearly hit a cyclist who has come up on your inside	-
In a queue of vehicles turning left, pay such close attention to traffic approaching from right	-
Drive back from a party, restaurant, or pub over the legal blood-alcohol limit	-
Have an aversion to a particular class of road user and indicate hostility	-
Lost in thought or distracted, fail to notice someone waiting at a zebra/pelican crossing	-
Park on a double-yellow line and risk a fine	-
Misjudge speed of oncoming vehicle when overtaking	-
Hit something when reversing that you had not previously seen	-
Fail to notice someone stepping out from behind a bus or parked vehicle	-
Plan your route badly and meet traffic congestion you could have avoided	-
Overtake a single line of stationary/slow vehicles, discover they were queuing	-
Overtake a slow-moving vehicle on the inside lane or hard shoulder of a motorway	-
Cut the corner on a right-hand turn and swerve violently to avoid an oncoming vehicle	-
Get into the wrong lane at a roundabout or road junction	-
Fail to read the signs correctly and exit from a roundabout on the wrong road	-
Fail to give way when a bus is signalling its intention to pull out	-
Ignore „give way‟ signs and narrowly avoid colliding with traffic having right of way	-
Fail to check your mirror before pulling out, changing lanes, turning	-
Attempt to overtake a vehicle that you hadn't noticed was signalling right	-
Deliberately drive the wrong way down a deserted one-way street	-
Disregard red lights when driving late at night along empty roads	-
Drive with only „half an eye‟ on the road while looking at map, changing cassette/radio	-
Fail to notice pedestrians crossing when turning into a side-street	-
Get involved in unofficial „races‟ with other drivers	-
Race oncoming vehicles for a one-car gap on a narrow/obstructed road	-
Brake too quickly on a slippery road and/or steer the wrong way in a skid	-
Misjudge your crossing interval when turning right and narrowly miss collision	-

Appendix B 

**Table 7:** Injury Severity Score (ISS) Developed by Baker et al. (1974) [12].

Items	Responses
Head and neck worst injury?	-
Face worst injury?	-
Chest worst injury?	-
Abdomen worst injury?	-
Extremity (including pelvis) worst injury?	-
External worst injury?	-
